# Pharmacokinetics-based adherence measures for antiretroviral therapy in HIV-infected Kenyan children

**DOI:** 10.7448/IAS.20.1.21157

**Published:** 2017-06-15

**Authors:** Wanzhu Tu, Winstone M Nyandiko, Hai Liu, James E Slaven, Michael L Scanlon, Samuel O Ayaya, Rachel C Vreeman

**Affiliations:** ^a^ Department of Biostatistics, Indiana University School of Medicine, Indianapolis, IN, USA; ^b^ Academic Model Providing Access to Healthcare (AMPATH), Eldoret, Kenya; ^c^ Department of Child Health and Paediatrics, School, Moi University, College of Health Sciences Moi University, Eldoret, Kenya; ^d^ Department of Pediatrics, Indiana University School of Medicine, Indianapolis, IN, USA

**Keywords:** pharmacokinetics, adherence, electronic dose monitoring, Nevirapine, measurement validation, pediatrics

## Abstract

**Background**: Traditional medication adherence measures do not account for the pharmacokinetic (PK) properties of the drugs, potentially misrepresenting true therapeutic exposure.

**Methods**: In a population of HIV-infected Kenyan children on antiretroviral therapy including nevirapine (NVP), we used a one-compartment model with previously established PK parameters and Medication Event Monitoring Systems (MEMS®)-recorded dosing times to estimate the mean plasma concentration of NVP (Cp) in individual patients during 1 month of follow-up. Intended NVP concentration (Cp’) was calculated under a perfectly followed dosing regimen and frequency. The ratio between the two (R = Cp/Cp’) characterized the patient’s NVP exposure as compared to intended level. Smaller R values indicated poorer adherence. We validated R by evaluating its association with MEMS®-defined adherence, CD4%, and spot-check NVP plasma concentrations assessed at 1 month.

**Results**: In data from 152 children (82 female), children were mean age 7.7 years (range 1.5–14.9) and on NVP an average of 2.2 years. Mean MEMS® adherence was 79%. The mean value of R was 1.11 (SD 0.37). R was positively associated with MEMS® adherence (*p* < 0.0001), and lower-than-median R values were significantly associated with lower NVP drug concentrations (*p* = 0.0018) and lower CD4% (*p* = 0.0178), confirming a smaller R value showed poorer adherence.

**Conclusion**: The proposed adherence measures, R, captured patient drug-taking behaviours and PK properties.

## Introduction

Adherence to antiretroviral therapy (ART) is an essential component of successful management of HIV/AIDS [1]. Studies have consistently shown strong associations between poor ART adherence and adverse clinical outcomes, including patient mortality [2], disease progression as measured by CD4 cell counts [3,4] and viral load [5–7], and development of drug resistance [8,9]. Despite mounting evidence on the benefit of ART adherence, suboptimal patient adherence remains common in adults and in children [10,11]. In addition, accurate and objective measurement of ART exposure, particularly for children in resource-limited settings (RLS), remains a challenge as adherence studies have used different adherence assessment methods and reported inconsistent findings [12].

A direct consequence of poor ART adherence is the lower-than-intended level of therapeutic exposure. Traditional medication adherence measures, such as patient self-reports, while easy to obtain, do not always have good concordance to actual adherence levels or clinical outcomes [13–15]. While measures like pharmacy refill records have performed better in some studies, their validity among children in RLS has been under-explored [16,17]. Moreover, most studies on adherence in resource-limited studies have not incorporated plasma drug concentrations and thus actual levels of exposure to ART has not been evaluated. Electronic dose monitors like Medication Event Monitoring Systems (MEMS®, MWV/AARDEX Inc., Switzerland) record the precise timing of medication bottle openings and thus may provide a more accurate record of a patient’s dosing events and medication taking behaviour. Indeed, ART adherence measures based on MEMS®, typically calculated as the ratio of bottle openings (i.e., “doses taken”) and the total number of doses prescribed for a given time period, are more strongly correlated with HIV virologic responses compared with other measures [15]. Still, the existing MEMS adherence measures do not account for the pharmacokinetic (PK) properties of the drugs and thus do not reflect patients’ true therapeutic exposure. It is generally understood that the PK properties of a medication can significantly affect the therapeutic outcome [18]. For example, drugs with longer half-lives tend be more forgiving of occasional dose omissions and also more tolerant of variations in dosing time [19].

The objective of the current research is to develop a new adherence measure that combines the recorded MEMS® dosing times with established PK parameters of Nevirapine (NVP) for quantification of patients’ NVP exposure. We approximated actual NVP exposure and compared it to the intended level of exposure in individual patients, potentially enhancing the extent to which MEMS® data can be used to understand whether patients reach therapeutic exposure levels. This is an approach that we have previously used to investigate patients’ adherence to beta-adrenergic antagonist metoprolol [20]. In the present study, we constructed a similar PK-based measure for quantification of patient adherence to NVP in cohort of HIV-infected Kenyan children.

## Methods

### Study design

This study is part of an ongoing investigation aimed at developing medication adherence assessment tools for HIV-infected Kenyan children. The study protocol has been described previously [21,22]. Briefly, HIV-infected children <14 years of age on first-line paediatric ART regimens who attended a large paediatric HIV clinic of the Academic Model Providing Access to Healthcare (AMPATH) in Eldoret, Kenya were followed prospectively with monthly clinical visits at which time various adherence assessments were administered. Informed consent was obtained from eligible participants and their caregivers prior to study enrolment. The study protocol was approved by the Institutional Review Board of Indiana University School of Medicine in Indianapolis, Indiana, and by the Institutional Research Ethics Committee at Moi University School of Medicine in Eldoret Kenya.

### Clinical assessment

At baseline, patients received clinical examination. Basic demographic and relevant clinical information was ascertained. As part of the study protocol, patients received NVP in MEMS® electronic dose monitors that created time stamps for all bottle openings. Patients were informed of the purpose of MEMS® and instructed in care of the cap and bottle. Dosing time data recorded by the MEMS® were read into PowerView software (Version 3.5.2; AARDEX, Inc.) and then converted into a SAS dataset for further analysis (Version 9.4; SAS Institute, Inc., Cary North Carolina). Adherence based on MEMS® bottle openings were calculated as a percentage using the number of doses taken (i.e., bottle openings) divided by the number of doses prescribed. A two-hour window was used to calculate doses taken – i.e., repeated openings within 2 h from the initial opening were not counted. This research was based on MEMS® data collected after 1 month of follow up. At 1 month of follow up, blood samples were collected from all patients for CD4 cell percentage (CD4%) and NVP drug concentration levels.

### Construction of PK-based medication adherence measures

We constructed ART adherence measures by estimating and contrasting the patient’s plasma concentration of NVP (Cp) to its intended levels (Cp’). We measured the ratio, or proportion, of intended therapeutic level achieved (R = Cp/Cp’). In this research, we approximated the hourly NVP concentrations via an iterative algorithm by a one-compartment model with MEMS®-recorded dosing times and previously established PK parameters [23].

The calculation was carried out with an iterative algorithm based on the standard 1-compartment model

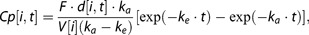

where 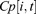 is the *i*th subject’s NVP concentration at time *t*, 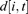 is the subject-specific dose level at time *t*, 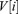 is the volume of distribution, and 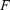 is the bioavailability of NVP. In the current study, we used 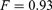, and 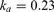/h, and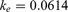/h. Dose 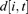 was determined based on the child’s age and weight [24]. With multiple dosing, we calculated the NVP concentration of a given subject at hourly interval. An iteratively algorithm was used to take into consideration of the cumulative effects of repeated dosing. Details of the algorithm were presented in the Appendix.

**Figure 1.**
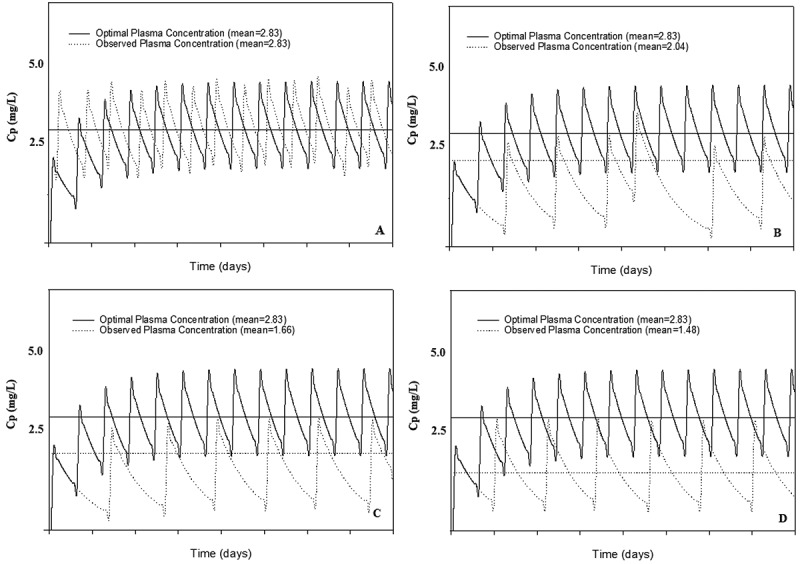
Observed versus optimal plasma concentrations using the PK-based measure in 4 paediatric patients. Curves plotted on graph with NVP drug concentration (y-axis) and time (x-axis). Observed (dotted line) versus intended (solid line) NVP plasma concentration curves are shown for 4 paediatric patients (clockwise from top left, patient A, B, C, D) with varying levels of adherence: A has good adherence (R = 1.066), B has fair adherence (R = 0.743), C has poorer adherence (R = 0.620), and D has very poor adherence (R = 0.565) (Seven days of data shown.).

The mean NVP concentration (Cp) was obtained by averaging the hourly NVP levels. Similarly, we calculated Cp’ by using the same PK parameters under the ideal medication taking behaviour (i.e., no missed doses and perfectly spaced dosing time). Specifically, we expressed the plasma concentration of NVP exposure for the *i*th patient at *t*th hour as 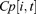, where 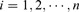, and 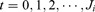,. Using a one-compartment model under a given set of PK parameters, we calculated 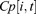 through an iterative algorithm, which incorporates the cumulative drug concentrations from previous doses taken based on the patient’s MEMS® records [20]. We calculated the intended NVP concentration for *i*th patient at *t*th hour as 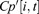 in a similar fashion, under the perfect dosing times. The estimated NVP levels (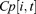) were graphically presented and visually compared to the intended NVP levels (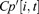) over time. To illustrate, data from 4 patients are presented in Figure 1.

Averaging the hourly drug concentration levels 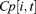 and 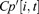, we obtained the estimated mean NVP levels under the observed dosing times, 
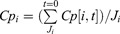
 and intended dosing times, 
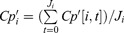
. We proposed to use 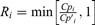 to characterize the patient’s level of medication adherence. Intuitively, 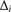 represented the average amount of the patient’s deviation from the intended plasma NVP concentration, whereas 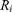 was the proportion of intended NVP concentration achieved under the recorded dose taking behaviour. The ratio 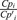 could exceed 1 if a patient opened the MEMS® lid more often than supposed to. To prevent spurious interpretation of 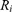 value great than 1, we capped 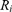 at 1.

It should be noted that the population PK parameters were unable to fully account for the observed between-subject variation. Factors at the patient level, such as route of administration, drug binding and local metabolism, characteristics of absorbing surfaces, and gastroenterological conditions may well affect the absorption and elimination of the drug. But introduction of patient-specific PK parameters would greatly reduce the practical utility of the proposed adherence measure, and thus defeating the purpose of the research. This said, the use of subject-specific volume distribution, dose level, and dosing times helps to accommodate, at least in part, the large variability that might exist across subjects. Finally, we noted that the estimation of NVP concentration at hourly intervals was proportional, and differed from the area under the fitted drug concentration curve (AUC) by a constant. The proposed measure, therefore, could be viewed as an effort to quantify drug exposure by comparing the AUCs under the intended and actually recorded dosing times.

### Measurement validation

We validated the proposed measure 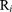 by evaluating its association with our primary end point, MEMS®-defined adherence, as well as to secondary end points CD4% and spot-check NVP plasma concentrations, all assessed at 1 month of follow up. Given the delayed impact of adherence on CD4%, we also validated the proposed measure against CD4% taken at 4 months of follow up (results not presented and were sufficiently similar to results at 1 month of follow up). Additionally, we calculated the correlation between the PK-based measure (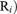 and compared the mean R levels in patients with ≥90% and <90% MEMS® adherence. We then assessed R’s association with the spot-check NVP concentrations as well as CD4% using regression analyses. We dichotomized the calculated R values at the sample median, and compared the spot check NVP concentration levels and mean CD4% values between those who have lower-than-median and higher-than-median R values, with these analyses being adjusted for age, sex, and ART duration. All analyses were performed using SAS software (Version 9.4, SAS Institute, Inc., Cary, North Carolina). *P*-values less than 0.05 were considered statistically significant.

## Results

### Demographic and clinical characteristics

We analyzed data from 152 children (82 female). Demographic and clinical characteristics of the study patients at baseline are presented in Table 1. At enrolment, mean age of the study patients was 7.7 years (standard deviation, SD = 3.2). Subjects were on NVP for an average of 2.2 years (SD 1.8 years). Children had moderate-to-severe clinical disease (57.8% were at WHO Stages 3 or 4) with mean CD4% of 27% at the study entry. At the 1 month follow-up visit, the subjects had an average MEMS® adherence of 79% (SD 26%), a mean CD4% of 27.6% (SD 10.2%), and a mean NVP concentration of 9.45 mg/L (SD 6.94 mg/L) (Table 2).

**Table 1.** Demographic and clinical characteristics of the study subjects

Cohort Characteristics N = 152	Mean (standard deviation) or Frequency (%)
*Child Characteristics*	
Mean age, years	7.7 (3.2)
Female	82 (56%)
Mean weight-for-age z score	−1.5 (1.2)*
Mean ART duration, years	2.2 (1.8)
Mean CD4%	27% (10%)
WHO stage	
1	30 (20%)
2	30 (20%)
3	75 (51%)
4	10 (7%)
Not answered	2 (1%)
*Caregiver and/or Family Characteristics*	
Caregiver relationship to child	
Mother	98 (67%)
Father	14 (10%)
Sibling	1 (1%)
Grandparent	7 (5%)
Non-relative	6 (4%)
Other	21 (14%)
Individuals who give the child ART**	
Mother	126 (83%)
Father	50 (33%)
Sibling	64 (42%)
Other relative	63 (42%)
Child took own	43 (28%)
Caregiver employed outside the home	119 (81%)
Enrolled in AMPATH nutrition program	25 (17%)
Food insecurity (reported “not enough food for family”)	100 (68%)
Reported difficulty with transportation to clinic	121 (82%)

*Weight-for-age Z scores based on World Health Organization Child Growth Standards

**More than one individual could be selected as giving ART to child

**Table 2.** Medication adherence and clinical outcome at one month (means and standard deviations)

MEMS® (% doses taken)	78.91 (26.43)
CD4%	27.65 (10.19)
NVP Spot Check	9.45 mg/L (6.94)
Cp	3.10 mg/L (1.43)
Cp’	2.83 mg/L (0.62)
R	1.11 (0.37)

The estimated and intended NVP exposure levels are presented in Table 2. Specifically, the mean values Cp, Cp’, and R value were 3.10 mg/L (SD 1.34 mg/L), 2.83 mg/L (SD 0.62 mg/L), and 1.11 (SD 0.37), respectively. The mean ratio of greater than 1 indicated that on average, the subjects in the study cohort adhered well to their prescribed medicine; some subjects had in fact opened their medication containers slightly more frequently than expected. Among all of the hourly estimated values of NVP concentration from all subject, approximately 45% were above the trough level of 3 mg/L.

### Validation of the PK-based adherence measures

Before validating the proposed adherence measures, we examined the estimated NVP concentration levels overtime in study subjects (data not shown). As illustrated by the four selected cases in Figure 1, the magnitude of 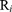 was strongly related to the MEMS® bottle opening patterns. While occasional omission of a dose did little to impact 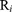, more systematic skipping of NVP doses resulted in significantly reduced 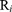 levels.

**Figure 2.**
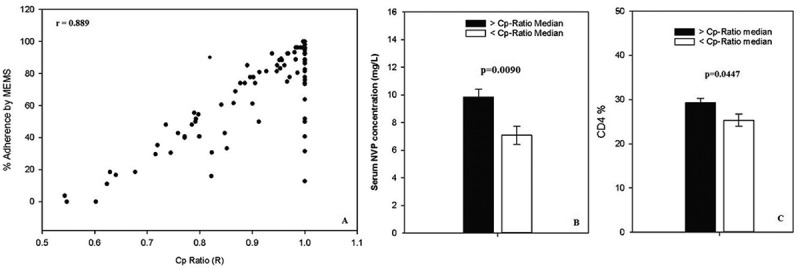
(a) Scatter plot of MEMS vs. R (left panel); (b) Spot check NVP concentration by R median categories (middle); (c) CD4% by R median categories (right).

The ratio R was positively associated with MEMS® adherence (Figure 2(b); 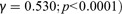. Patients with lower-than-median R values had significantly lower NVP concentration than those who had higher-than-median R values (Figure 2(c), 7.546 vs. 9.835 mg/L; *p* = 0.0090), as measured in the spot-check samples. Patients with lower-than-median R values also had significantly lower CD4% (Figure 2(d); 25.97% vs. 29.29%; *p* = 0.0447). Correlation analysis also confirmed that a lower R value was also associated with lower spot-check plasma concentration (*p* < 0.0001), but R’s association with CD4% did not reach the level of statistical significance (*p* = 0.2718).

These findings confirmed our hypothesis that a smaller R value was associated with non-adherence, as measured by both MEMS® adherence and by spot-check NVP concentration; they were also associated with significantly reduced CD4% in study subjects.

## Discussion

In this research, we proposed and validated a novel PK-based measures for NVP adherence in Kenyan children. The proposed measure, R, differs from existing adherence measures (e.g., MEMS® monitoring alone) in its ability to accommodate the PK properties of the drug, and to characterize patient adherence by comparing the estimated drug exposure under MEMS-recorded dosing times to that obtained under the perfectly followed dosing times. In other words, our measure accounted not only for patient’s drug taking behaviour but also the properties of the drug, which makes it a more clinically useful measure of adherence to medication. While patient’s drug-taking behaviour is a major contributor to the therapeutic exposure, behaviour alone does not solely determine the level of exposure. The accommodation of the PK properties of the drug incorporates a previously unaccounted factor into consideration. In addition, with the iterative algorithm for 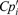 and 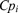, occasional dose omission and systematic dosing time shifting were appropriately incorporated into the calculation. In a sense, 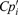 and 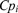 could be viewed as approximation of the area under the drug concentration curves under the ideal and actual dosing times. In this regard, the standard MEMS® adherence measure ignores the effect of actual dosing times, and thus under-utilized collected dosing event data. Together, the measure offered an appealing set of characteristics that made it suitable for monitoring NVP adherence and exposure simultaneously – even when one does not have access to drug concentration data. While these measures require established PK drug parameters and precise dose timing records, they may offer great potential for controlled trials and drug studies that require more accurate drug exposure estimates over prolonged periods of time.

There are several unique advantages to R. The measure, R, which represents the average percentage of drug levels achieved by the patent, is a unitless measure because it is based on the intended and actual drug levels. While the calculation of R requires PK parameters, the adherence measure, R, as a ratio of intended and actual is unitless and thus not drug specific. In addition to being easy to interpret, the measure, R, could be used to comparatively assess adherence levels across patients with varying drug doses and also across drugs. In most clinical settings, we contend, R is likely to be a practical measure to use. In our findings here, R values were somewhat greater than MEMS-measured adherence, suggesting that NVP is not hugely sensitive to dose timing.

This study has a number of important limitations to consider. First, as noted earlier, one of the major limitations of R as an adherence measure is that it requires established population PK parameters. As a result, the estimated concentration curves will not fully account for the between subject variations. To some extent, the use of subject-specific doses and the precisely recorded dosing times might help to better accommodate the potentially large variability in drug concentration, as drug absorption and elimination are known to be related to local conditions of drug metabolism. But for children in a RLS, particularly in sub-Saharan Africa, there are few PK data for major antiretroviral drugs, introducing subject-specific PK parameters, or treating them as functions of observed patient factors would greatly increase the difficulty of computation, severely limit the potential for the practical use of the proposed measure. The reliance on PK data also restricts the measures’ generalizability. In addition, dose-timing technology remains relatively expensive although there are reasons to believe that the cost of MEMS® could substantially decrease in the coming years given the sustained interest in implementing adherence technologies for routine clinical use. For the same reason, we did not consider drug-to-drug interactions in the current study; those interactions may affect the metabolism of the NVP. Second, the current research could not validate R against carefully assessed viral loads or viral resistance patterns, which are generally viewed as the most significant clinical endpoints for adherence to ART but are routinely not available in RLS such as western Kenya. We tried to overcome this limitation by validating our adherence measure against three available end points (MEMS®, CD4%, and spot-check drug concentration), which revealed consistent results of the measures’ validity. Finally, we relied on MEMS® for records of dosing time, but of course we could not be sure that the patient did actually take the medicine each time he/she opened the MEMS® bottle (and vice versa, we did not know if they took out more than one dose). In previous work, we have shown high correlation between MEMS® adherence and CD4% and spot-check drug concentrations, as well as caregiver-reported missed doses, suggesting that MEMS® reflects actual drug taking behaviour in this cohort [21]. Notwithstanding these limitations, we contend that the measure described in this paper represent a novel attempt to more objectively quantify the patients’ exposure to a prescribed therapeutic agent and provides a methodology that should be tested through more rigorous evaluation that includes viral load and genotypic resistance testing.

## Conclusions

We created and validated a novel adherence measure that accommodates the PK properties of the drug and medication taking behaviours. This measure offers key advantages to traditional medication adherence measures, particularly in research settings where more precisely measured drug exposure is needed but direct assessment is not feasible.

## Notes

To access the supplementary material to this article please see Supplementary Files under Article Tools online.
